# Supplementary material for: Photolysis of Fluorinated Graphites with Embedded Acetonitrile Using a White-Beam Synchrotron Radiation
Source: Nanomaterials (Basel). 2022 Jan 11;12(2):231. doi: 10.3390/nano12020231 (PMC8779973; doi:10.3390/nano12020231)
Supplement: Supplementary file 1 [file nanomaterials-12-00231-s001.zip › nanomaterials-1510364-supplementary.pdf]

nanomaterials

MDPI

Supporting information for

Article

Photolysis of Fluorinated Graphites with Embedded Acetonitrile Using a White-Beam Synchrotron Radiation

Galina I. Semushkina [1,*], Yuliya V. Fedoseeva [1], Anna A. Makarova [2], Dmitry A. Smirnov [3], Igor P. Asanov [1], Dmitry V. Pinakov [1], Galina N. Chekhova [1], Alexander V. Okotrub [1] and Lyubov G. Bulusheva [1,*]

[1] Nikolaev Institute of Inorganic Chemistry SB RAS, 3 Acad. Lavrentiev Ave., 630090 Novosibirsk, Russia; fedoseeva@niic.nsc.ru (Y.V.F.); asan@niic.nsc.ru (I.P.A.); pinakov@niic.nsc.ru (D.V.P.); chekhova@niic.nsc.ru (G.N.C.); spectrum@niic.nsc.ru (A.V.O.)

[2] Physikalische Chemie, Institut für Chemie und Biochemie, Freie Universität Berlin, 14195 Berlin, Germany; anna.makarova@fu-berlin.de

[3] Institut für Festkörper- und Materialphysik, Technische Universität Dresden, 01069 Dresden, Germany; dmitry.smirnov@helmholtz-berlin.de

* Correspondence: semushkina.g@niic.nsc.ru (G.I.S.); bul@niic.nsc.ru (L.G.B.)

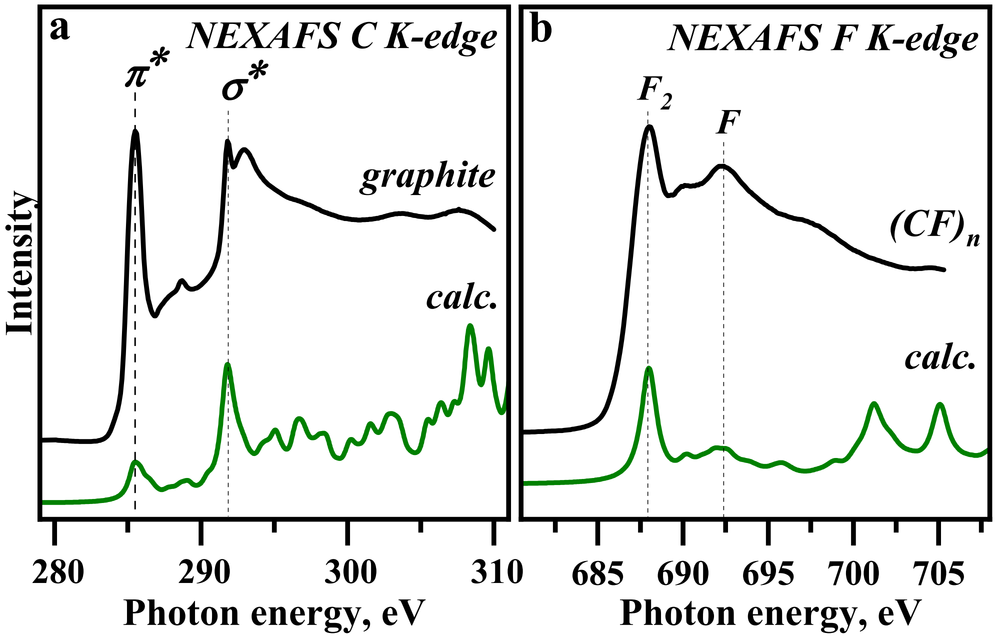

Figure S1. Experimental NEXAFS C K-edge spectrum of graphite (a) and F K-edge spectrum of $(CF)_n$ (b) in comparison with the corresponding theoretical spectra, constructed for nitrogen atom, which replaces the central carbon atom in the graphene $C_{96}F_{24}$ model, (a) and for neon atom, which replaces the central fluorine atom in the fully fluorinated graphene $C_{96}F_{86}$ model, (b) within the (Z + 1)-approximation. The theoretical C K-edge spectrum is aligned to the experimental spectrum by positions of π* and σ* resonances and this gives $E = 0.86 \cdot E_{calc} - 50.8$ eV for scaling the energy scale. The theoretical F K-edge spectrum is aligned to the experimental $(CF)_n$ spectrum by peaks F_2 and F of the experimental spectrum and this gives a scaling formula E = 0.84·Ecalc + 128.5 eV.

Table S1. Binding energy (BE) and relative area (A) of the components in the XPS C 1s spectra of $CH_3CN@CF_{0.3}$ and $CH_3CN@CF_{0.5}$ samples before and after exposure to non-monochromatized SR light for 80 and 200 s.

Exposure time, s	Components	$CH_3CN@CF_{0.5}$		$CH_3CN@CF_{0.3}$	
		BE (eV)	A (%)	BE (eV)	A (%)
0	C (sp^2)	284.5	8	284.5	21
	C–CF	286.1	45	285.4	47
	C–N	287.0	2	287.0	5
	C–F	288.7	43	288.0	24
	sat.	292.0	2	291.0	3
80	C (sp^2)	284.5	9	284.5	52
	C–CF	285.2	54	284.9	22
	C–N	286.5	6	286.2	8
	C–F	287.8	27	287.5	12
	CF_2	289.5	3	289.0	3
	CF_3	292.0	1	291.0	3
200	C (sp^2)	284.5	25	284.5	59
	C–CF	285.2	39	284.9	19
	C–N	286.5	4	286.2	5
	C–F	287.8	18	287.5	10
	CF_2	289.5	8	289.0	4
	CF_3	292.0	6	291.0	3

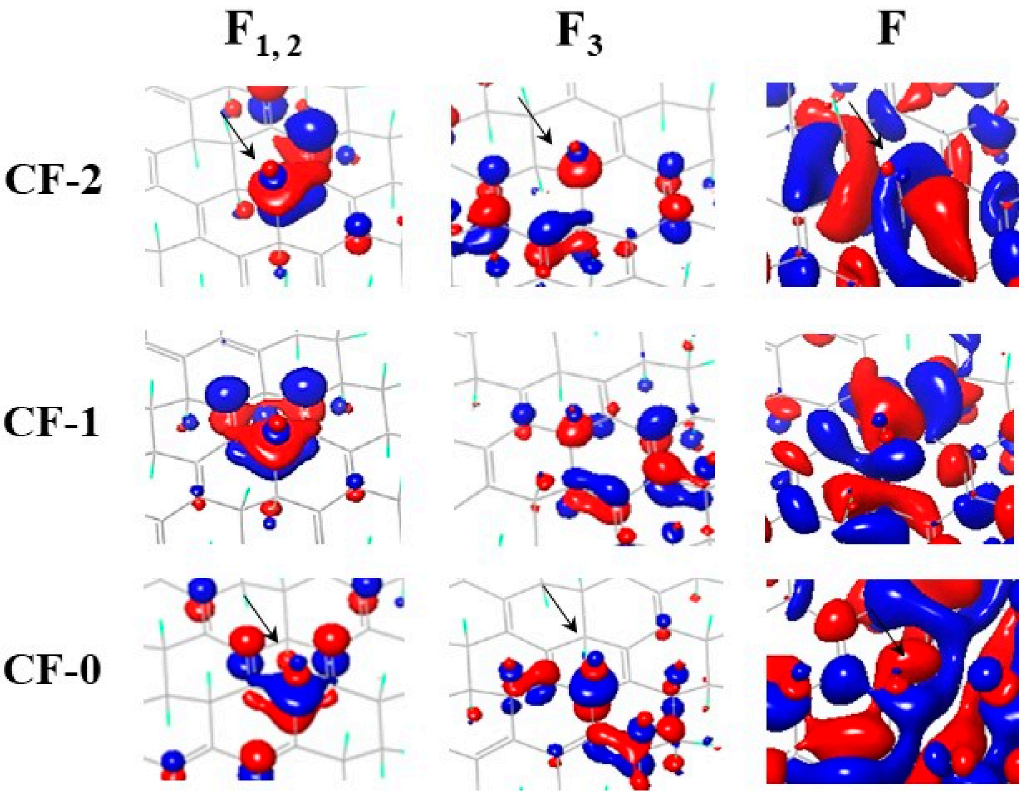

Figure S2. Unoccupied molecular orbitals (MOs) of fluorinated graphene fragments $(C_{96}F_{85}Ne_1)^+$ calculated in the (Z + 1)-approximation with Ne atoms replaced structurally nonequivalent fluorine in CF groups surrounding by two (CF-2), one (CF-1) and none (CF-0) neighboring CF groups. Arrows indicate the position of Ne atoms. The density of states for the MOs responsible for F_1, F_2, F_3, and F features in the calculated NEXAFS F K-spectra of CF-2, CF-1 and CF-0 are plotted. Isosurfaces of positive (blue) and negative (red) wave functions were modeled with coefficient -0.05 for F_1, F_2, F_3 molecular orbitals and -0.02 for F.

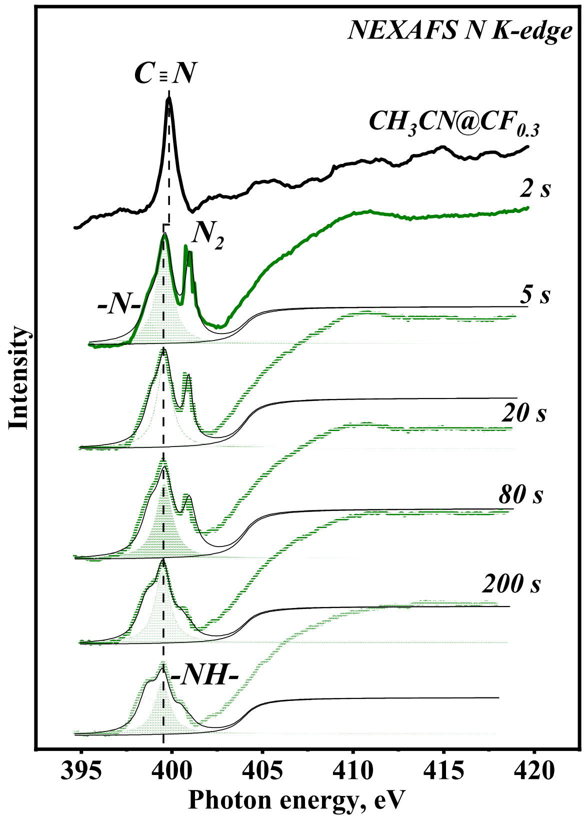

Figure S3. NEXAFS N K-edge spectra of $CH_3CN@CF_{0.3}$ before and after irradiation for 2, 5, 20, 80 and 200 s. π*-states in the spectra of irradiated sample are fitted by Lorentz-shape lines corresponding to pyridinic N (–N–), HC≡N, pyrrolic N (–NH–), and N_2.

Table S2. Binding energy of a fluorine atom located at the edge or in the center of fluorinated region $C_{150}F_{30}F_z$ where z = 2, 14, 24 in the models shown in Figure S4. The models were calculated using B3LYP/ DEF2-SVP in the ORCA 4.2.1 program.

Region	E, eV	
	on the edge	in the center
$C_{150}F_{30}F_2$	3.0	-
$C_{150}F_{30}F_{14}$	2.4	4.1
$C_{150}F_{30}F_{24}$	3.8	5.1

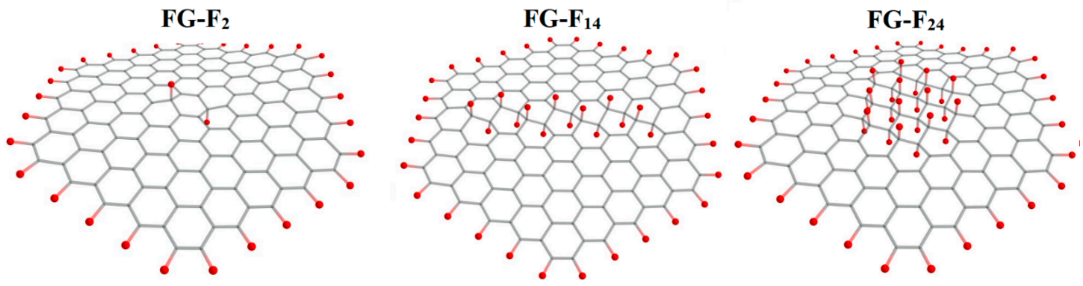

Figure S4. Optimized fluorinated graphene fragments $C_{150}F_{30}F_{z}$ (z = 2, 14, 24), where fluorine atoms are attached to the basal plane as a pair (FG-F_2), zigzag chains F_{14} and with the formation of fluorinated cluster area (FG-F_{24}).

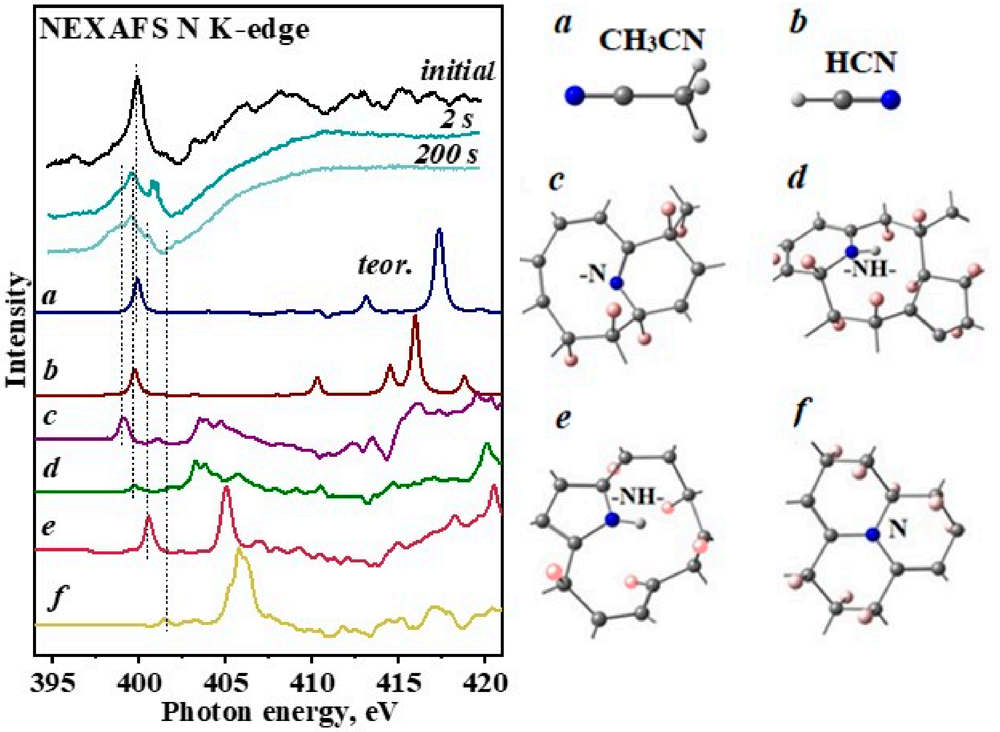

Figure S5. Experimental NEXAFS N K-edge spectrum of $CH_3CN@CF_{0.5}$ before and after irradiation for 2 and 200 s (top curves) and theoretical NEXAFS N K-edge spectra of CH_3CN (a) and HCN (b) molecules and nitrogen atoms inserted into the fluorinated graphene fragment in the form of pyridinic (c), hydrogenated pyridinic (d), pyrrolic (e) and graphitic nitrogen atoms (f).

NEXAFS N K-edge spectra for CH_3CN, HCN molecules, pyridinic N and hydrogenated pyridinic N in $C_{92}F_{85}N_2H$ model, pyrrolic N in $C_{93}F_{84}NH$ model, and graphitic N in $C_{95}F_{86}N$ model were calculated using the (Z+1)-approximation in the ADF 2020 program [58]. The calculations were performed at the B3LYP/TZP level. For the model calculation, the SCAN functional was used, which has shown its efficiency for similar nitrogen-containing systems [59]. Geometry optimization was performed using the default convergence criteria. The absence of imaginary frequencies indicated that the resulting structures corresponded to the local minimum on the potential energy surfaces.
